# The Trends and Future Prospective of In Silico Models from the Viewpoint of ADME Evaluation in Drug Discovery

**DOI:** 10.3390/pharmaceutics15112619

**Published:** 2023-11-12

**Authors:** Hiroshi Komura, Reiko Watanabe, Kenji Mizuguchi

**Affiliations:** 1 University Research Administration Center, Osaka Metropolitan University, 1-2-7 Asahimachi, Abeno-ku, Osaka 545-0051, Osaka, Japan; 2 Institute for Protein Research, Osaka University, 3-2 Yamadaoka, Suita 565-0871, Osaka, Japan; reiko-watanabe@protein.osaka-u.ac.jp (R.W.); kenji@protein.osaka-u.ac.jp (K.M.); 3 Artificial Intelligence Centre for Health and Biomedical Research, National Institutes of Biomedical Innovation, Health, and Nutrition (NIBIOHN), 3-17 Senrioka-shinmachi, Settu 566-0002, Osaka, Japan

**Keywords:** in silico model, ADME, artificial intelligence, machine learning, prediction, academic drug discovery

## Abstract

Drug discovery and development are aimed at identifying new chemical molecular entities (NCEs) with desirable pharmacokinetic profiles for high therapeutic efficacy. The plasma concentrations of NCEs are a biomarker of their efficacy and are governed by pharmacokinetic processes such as absorption, distribution, metabolism, and excretion (ADME). Poor ADME properties of NCEs are a major cause of attrition in drug development. ADME screening is used to identify and optimize lead compounds in the drug discovery process. Computational models predicting ADME properties have been developed with evolving model-building technologies from a simplified relationship between ADME endpoints and physicochemical properties to machine learning, including support vector machines, random forests, and convolution neural networks. Recently, in the field of in silico ADME research, there has been a shift toward evaluating the in vivo parameters or plasma concentrations of NCEs instead of using predictive results to guide chemical structure design. Another research hotspot is the establishment of a computational prediction platform to strengthen academic drug discovery. Bioinformatics projects have produced a series of in silico ADME models using free software and open-access databases. In this review, we introduce prediction models for various ADME parameters and discuss the currently available academic drug discovery platforms.

## 1. Introduction

The major goal of pharmaceutical research and development is to ensure the continuous availability of new chemical molecular entities (NCEs) which display a high therapeutic efficacy with few or no adverse effects. Efficacy and toxicity are associated with pharmacokinetic profiles governed by absorption, distribution, metabolism, and excretion (ADME). Consequently, poor ADME profiles of NCEs can be a major cause of attrition in drug development [1]. Major challenges in drug discovery include the chemical constraints derived from receptor or target ligands, and ADME plus toxicology (ADMET) properties are regarded as secondary constraints in the drug discovery process. ADME screening has become increasingly important in identifying and optimizing lead structures. Each ADME process can be further evaluated using certain parameters, i.e., absorption is evaluated mainly using solubility and membrane permeability; distribution using protein binding, tissue binding, and transporters such as P-glycoprotein (P-gp), which is a key factor in distribution to the central nervous system (CNS); metabolism using metabolic stability in liver microsomes or hepatic clearance; and excretion using renal clearance and urinary excretion rates. Many screening systems have been developed and applied to evaluate compounds based on the appropriate criteria during lead identification and optimization. To streamline these stages, ADME prediction using in silico models has become essential, and is ideally used to extract compounds for high-throughput screening from vast compound libraries or to guide structural design before chemical synthesis [2,3].

The most important challenge for in silico ADME screening includes setting up robust models with a high predictability. Recent model construction studies have predominantly utilized artificial intelligence (AI) and machine learning (ML) technologies, including artificial neural networks (ANN), random forests (RF), support vector machines (SVM), and tree-based methods [3]. Furthermore, current state-of-the-art technologies, such as graph convolution networks (GCNs) and graph neural networks (GNNs), have permitted researchers to generate novel in silico ADME prediction models in addition to assessing the biological activities of NCEs toward druggable protein targets [4,5]. These models can be divided into two types, classification models (that can select compounds based on cut-off values for each parameter) and regression models (that can provide the exact value). 

In silico ADME models are likely to be constructed by players in three major areas: information technology (IT), pharmaceuticals, and academia. IT companies have created predictive platforms for various ADME parameters, including toxicological profiles, such as ADMET Predictor (Simulations Plus, Lancaster, CA, USA; http://www.simulations-plus.com/ (accessed on 7 November 2023)), BIOVIA Discovery Studio (Accelrys, San Diego, CA, USA; https://www.3ds.com/products-services/biovia/products/ (accessed on 7 November 2023)), and SCIQUICK (Fujitsu Ltd., Tokyo, Japan; https://www.fujitsu.com/jp/solutions/business-technology/tc/sol/sciquick/ (accessed on 6 November 2023)). These platforms have emerged as powerful tools for drug discovery in the pharmaceuticals industry. Furthermore, effort has been made by different pharmaceutical companies to construct in-house ADME prediction platforms using datasets based on proprietary chemical libraries and data derived from in-house screening systems with optimized assay conditions. The complementary use of in-house platforms and commercially available suites allows for improved ADME screening and the efficient acceleration of NCE discovery.

It is interesting to note that basic academic research has garnered attention as an alternative method for discovering NCEs, and more than half of the recent small-molecule discoveries have their origins in academic research, with increasing relative inventive contributions from academia [6,7]. However, most academia-led drug discovery projects fail in the nonclinical stage (the so-called “death valley”). This is attributed to the lack of expertise and resources in academia to optimize lead structures in terms of their pharmacokinetics and toxicology. Academic researchers have limited access to commercial ADME prediction software packages due to high licensing fees; therefore, the establishment of a favorable research environment for such prediction tools is essential. 

To meet social demand, freely available prediction models have been developed, including online chemical modeling environments, such as OCHEM (https://ochem.eu/home/show.do (accessed on 6 November 2023)) [8], SwissADME (http://www.swissadme.ch/ (accessed on 6 November 2023)) [9], and pkCSM (http://biosig.unimelb.edu.au/pkcsm/ (accessed on 6 November 2023)) [10]. The Japan Agency for Medical Research and Development (AMED) directs integrated research to translate basic research into practical applications (https://www.amed.go.jp/en/ (accessed on 6 November 2023)). The Department of Innovative Drug Discovery and Development (iD3) division of AMED, which supports academic drug discovery, established the Initiative Development of a Drug Discovery Informatics System (iD3-INST) with financial support. The objective of the iD3-INST was mainly to construct a platform for academic drug discovery, which would comprise a database and in silico prediction models for ADME profiles. Attempts have been made in the public sector and in public–private partnerships to overcome the issues described above [11].

Given that pharmacokinetic behavior is governed by many parameters, an understanding of pharmacokinetics requires a comprehensive evaluation of these parameters. Many in silico models have been reported for each parameter. It is necessary to recognize the significance of these parameters when considering which model is appropriate for screening purposes. While this may be possible for pharmacokinetic researchers, medicinal chemists working in industry and academic biologists often do not have an in-depth understanding of these parameters. Therefore, this review describes the background and significance of evaluating each parameter in pharmacokinetic studies and then introduces the characteristics of various in silico models. Thus, this review will serve as a guide for ADME-based in silico drug discovery for researchers in various fields. The features of the open-access in silico models developed using the iD3-INST are also described. Toward the end, we briefly discuss the ongoing challenges with newly available AI technologies built by industry–academia partnerships, and the future prospective for in silico ADME prediction.

## 2. Evaluation Metrics of Predictive Models

We evaluated the predictive models more frequently cited in previous studies. A description of the evaluation metrics is shown in the tables, and their descriptions are given below.

- Classification model evaluation metrics

“Accuracy” measures the proportion of samples correctly classified by the model; however, it may not be suitable for imbalanced datasets. “Precision” calculates the ratio of true positive predictions to all positive predictions made by the model; it is important to minimize false positives. “Recall” calculates the ratio of true positive predictions to all actual positive samples; it is important to minimize false negatives. “F1” is the harmonic mean of precision and recall, and balances the trade-off between these two metrics. In addition, it is useful for assessing the overall model performance. “Receiver operating characteristic area under the curve (ROC-AUC)” quantifies the area under the ROC curve, which plots the true positive rate against the false positive rate at different thresholds. “Cohen’s kappa (kappa)” measures the agreement between observations, and it takes into account the possibility of agreement occurring by chance. It is a credible and dependable indicator of inter-rater agreement.

These metrics (other than kappa) are expressed in the range of 0–1; a higher value is desirable. The range of possible values of kappa is from −1 to 1, though it usually falls between 0 and 1.

b. Regression model evaluation metrics:

“Mean absolute error (MAE)” is the average of the absolute differences between the actual and predicted values. It quantifies the size of prediction errors without squaring the values and is less sensitive to outliers. “Mean squared error (MSE)” is the average of the squared differences between the actual and predicted values. It quantifies the size of prediction errors and is more sensitive to outliers than the MAE. “Root mean squared error (RMSE)” is the square root of the MSE, providing a similar measure of prediction error with the same units as the target variable. “Coefficient of determination (R^2^)” and “Cross-validation coefficient of determination (Q^2^)” represent the proportion of variation in the dependent variable that can be explained by the model, where Q^2^ is calculated based on a full cross-validation. R^2^ and Q^2^ provide a measure of how well the observed outcomes are replicated by the model, based on the proportion of total variation in the outcomes that can be explained by the model.

All the metrics in regression models take values from 0 to 1. Lower values of MAE, MSE, and RMSE and higher values of R^2^ and Q^2^ indicate a better model performance.

## 3. Physicochemical Properties

When considering the relationship between physicochemical properties and ADME, the most fundamental properties include lipophilicity, solubility, ionization, topology, and molecular weight (MW). In the subsequent subsections, we describe the definition, importance in ADME, and determination of each of these properties. 

### 3.1. Lipophilicity

Lipophilicity refers to the ability of a compound to interact with non-polar solvents and is a fundamental property for describing hydrophobicity.

It has been associated with the ADME, toxicity, and efficacy of NCEs and has been widely exploited when constructing in silico models for ADMET as a chemical descriptor. Increasing the lipophilicity of compounds via chemical modifications tends to enhance their penetration through cell membranes and biological barriers, in addition to their binding to proteins such as serum albumin, but reduces their aqueous solubility.

Lipophilicity is typically defined by the partition coefficient (P), which is determined based on the ratio of the concentrations of a solute between the two solvent phases (generally, with water and n-octanol). The logarithm of this ratio (log P) has also been utilized to indicate lipophilicity.

The distribution coefficient, log D, is the ratio of the sum of the concentrations of all forms of the compound (ionized plus unionized forms) in each of the two solvents (aqueous phase at a specified pH and n-octanol). Therefore, log D depends on the ionization of the compound and is equal to log P for non-ionizable compounds. For ionizable compounds, log D is preferentially utilized over log P when considering the relationship between lipophilicity and ADME. 

### 3.2. Solubility

Solubility refers to the ability of a compound to dissolve in a specific solvent (i.e., water or buffer with a specific pH, fasted state simulation intestinal fluid, and fed state simulated intestinal fluid). The solubility of a compound varies greatly depending on its salt and crystal structures and is also influenced by chemical structure, temperature, and the nature of the solvent. 

Solubility is a critical property in drug development because it directly affects bioavailability. Solubility is also an indicator of absorbability. For poorly soluble compounds, much effort is often needed to improve their intestinal absorption in the nonclinical stages (pharmacokinetic and toxicological studies) and to develop appropriate formulations for clinical trials. 

Solubility can be determined using the shake-flask method. In this method, a compound is added to a known volume of the solvent, and the mixture is shaken until equilibrium is reached. The concentration of the dissolved compound is then determined via various analytical techniques, such as high-performance liquid chromatography. 

### 3.3. Ionization

Ionization (indicated using the ionization constant (pKa)) is a measure of the acidity or basicity of a compound, specifically its ability to donate or accept protons (H^+^ ions) when in solution. 

pKa is an important parameter because it helps to explain the pharmacokinetic behavior and interactions of a compound. It is determined by measuring the pH at which the concentrations of the compound’s ionized and unionized forms are equal. When log D is measured at various pHs, a curve is drawn (based on a plot of log D against pH). The pH at the inflection point is the pKa, and log D is closely related to pKa.

### 3.4. Topology

Topology refers to the arrangement or connectivity of atoms in a molecule, specifically in the context of its chemical structure and the spatial relationships between atoms.

It is a fundamental concept in medicinal chemistry, and the connectivity and arrangement of atoms in a compound are key factors in determining its chemical and biological properties. Medicinal chemists often design the topology of a compound to optimize its binding affinity to specific targets and improve its pharmacokinetic properties.

### 3.5. Molecular Weight

The MW of a compound, also known as the molecular mass or molar mass, is the sum of the atomic weights (or atomic masses) of all the atoms in a molecule and is typically expressed in Daltons (Da).

A compound’s MW is a determinant of its ADME profile, and affects its membrane permeability; this consequently affects its intestinal absorption and tissue distribution, particularly in the context of penetration through the blood–brain barrier (BBB).

To calculate the MW, the atomic weights of all the atoms in the chemical formula are added up. Accurate MWs can be obtained using analytical instruments such as mass spectrometers.

## 4. Oral Absorption

### 4.1. General Assessment

Oral absorbability, alongside hepatic metabolic stability, is a key criterion for selecting NCEs with desirable pharmacokinetic properties [12]. Solubility and membrane permeability are determinants of oral absorption [13], and the Biopharmaceutics Drug Classification System has identified permeability and solubility as key parameters controlling absorption [14]. The significance of these two parameters can be demonstrated by the fact that the maximum absorbable dose is mainly dependent on water solubility and absorption rate constants, when the intestinal water volume is fixed [14,15]. A poor water solubility and membrane permeability profiles are challenges when selecting the appropriate solvents to achieve sufficient exposure in toxicokinetic studies and when developing formulations for clinical studies; these barriers can result in increased failure rates [16,17].

The terms “kinetic solubility” and “thermodynamic solubility” are often used to describe water solubility, and solubilities are assessed during several stages in drug discovery [18]. The kinetic solubility method, which utilizes a relatively high-throughput system, is determined after the addition of a small volume of DMSO to the aqueous buffer; this assay is widely considered to be the standard method [19]. Kinetic solubility values are typically higher than corresponding thermodynamic solubility values because supersaturation occurs when an organic solvent is diluted in water. Thermodynamic solubility is determined by dispensing a solid compound in a solvent. This is often considered to be the true solubility of the compound and is critical for formulation development [20]. The analysis of thermodynamic solubility has become streamlined recently through the development of miniaturized high-throughput assays in conjunction with novel analytical techniques, such as solid-state analysis, ultra-performance liquid chromatography, and polychromatic turbidimetry. Newly developed thermodynamic solubility assays enable more complex physicochemical profiling during drug discovery.

Cell-based models have been used to predict in vivo intestinal drug permeability by utilizing Caco-2 and other epithelial cell lines, such as human primary intestinal cells and induced pluripotent stem cells [21]. Caco-2 cells, which are derived from human colorectal adenocarcinoma cells, have been widely used by pharmaceutical companies and regulatory authorities as a standard assessment line, and have been widely used for in vitro cell culture models of the human intestinal mucosa. The Caco-2 cell assay commonly uses a 24-well format with a long-term culture, but is considered to be labor-intensive, time-consuming, expensive, and low throughput. The 24-well assay plate format has been miniaturized to a 96-well assay format [22,23]. As alternative approaches, a short-term growing-cell assay with Madin–Darby canine kidney (MDCK) cells and parallel artificial membrane permeability assays (PAMPAs) have been used for permeability screening in drug discovery [24,25]. Given that wild-type (WT) MDCK cells are genetically heterogeneous, Bokulic et al. [26] developed an assay system for assessing passive permeability based on a subclone of MDCK cells, which lowly expressed P-gp. Consequently, in silico permeability prediction models have been developed in accordance with various assay methods.

Several in silico models for solubility, membrane permeability, and intestinal absorption have been developed, and Table 1 summarizes the characteristics of some of these models. 

### 4.2. Solubility Prediction

Most available in silico solubility predictors are classification models rather than quantitative structure–activity relationship (QSAR) models. Newby et al. [27] developed in silico models with binary classifiers using 483 compounds obtained from the AQUASOL (6th Edition) and SRC (PHYSPROP) databases, and the model achieved a sensitivity and selectivity of 0.823 and 0.879, respectively. Siramshetty et al. [28] investigated the ability of QSAR and classification models to predict ADME properties, such as metabolic stability, permeability, and solubility; an in silico prediction model for solubility was constructed based on kinetic solubility data from a large dataset of 25,853 compounds. Binary classification models with a cut-off of 10 μg/mL were built using RF and graph convolutional neural networks (GCNN), and achieved a relatively high predictive performance with an AUC-ROC curve of 0.87–0.90, sensitivity of 0.63–0.71, and specificity of 0.90–0.91. Falcón-Cano et al. [29] suggested that the limited performance of in silico solubility models may be attributable to the lack of high-quality solubility data. Their group developed a QSAR model and a binary classification model based on a large and diverse dataset including thermodynamic solubility information collected from two public sources in accordance with the curation protocol, i.e., including the cleaning of the chemical structures, standardization of the molecular representation, and treatment of duplicates. The consensus model for the QSAR and binary classification models with cut-offs of log S > 10^−2^ produced a coefficient determinant (R^2^) of 0.87 and sensitivity and specificity of 0.96 and 0.80, respectively, for the internal test set. It should be noted that their groups intensively summarized existing in silico classification and QSAR models. A novel prediction approach for biorelevant solubility was recently reported, which was based on a thermodynamic cycle comprising solid-state crystal lattice packing and the dissociation of molecules from the lattice and solvation [36]. This approach provides important information on the molecular interactions that occur during drug dissolution and solubilization.

### 4.3. Prediction of Membrane Permeability

The Caco-2 cell assay is a standard method for evaluating the membrane permeability of molecules in the pharmaceutical industry. Large variabilities in membrane permeability (*P*_app_) predictions between laboratories, which can be attributable to different assay conditions and/or heterogenous cell lines [22], have become a common issue. Although reference compounds (i.e., metoprolol) have been utilized to validate this routine assay, data correction based on the *P*_app_ of reference compounds is necessary. Therefore, it is difficult to develop QSAR models with a high accuracy using large datasets comprising *P*_app_ data collected by various laboratories. Smaller datasets tend to be used when constructing in silico models based on Caco-2 cells to predict permeability. An attempt utilizing an in-house dataset by Kamiya et al. [30] yielded QSAR models via multivariant linear regression, but the predictive performance was relatively low with a correlation coefficient (R) of 0.77. Ta et al. [31] curated permeability data by evaluating Caco-2 cell assay conditions, such as the pH and solvent used, to ensure consistency. Using the curated data, the QSAR models built with innovative ML-based hierarchical support vector regression (HSVR) achieved a high correlation (R^2^ = 0.91). Brocatelli et al. [32] employed MDCK data to build a binary-classifier model, and the predictive performance based on AUC was 0.84 for partial least-squares regression (PLSR) and 0.81 for SVMs. Noticeably, the *P*_app_ originating from the Caco-2 cell assay was correlated with that from the MDCK cells, with an R^2^ of 0.60.

PAMPA is widely utilized for early screening in drug discovery due to its simple mechanism, resulting in the development of in silico QSAR models. QSAR models for PAMPA developed by Nakao et al. [34] and Chi et al. [35] showed similar predictive performances with an R^2^ of 0.76 and a Q^2^ of 0.61–0.88, respectively, despite the small datasets used. Siramshetty et al. [28] updated PAMPA QSAR models using data generated through an in-house optimization process, and achieved a balanced accuracy of 71–85%. Additionally, their group developed a binary-classifier based on a cut-off of 2.5 for *P*_app_ using a large dataset of 16,624 compounds, which achieved a good predictive performance with an AUC-ROC of 0.85–0.86, sensitivity of 0.83–0.84, and specificity of 0.64–0.78. Similarly, a relatively large dataset (>6500) for PAMPA was employed by Williams et al. [24], who demonstrated the usefulness of a GCNN-developed four-classifier model and good correlation between PAMPA permeability at pH 5 and in vivo oral bioavailability in mice and rats.

### 4.4. Prediction of Intestinal Absorption 

Several attempts have been made to identify the molecular descriptors that are associated with intestinal absorption as well as membrane permeability in cell-based assays [37,38,39]. A review by Stenberg et al. [37] reported a chemical descriptor-based method for prediction of absorption in humans and found that the polar surface area (PSA) was closely related to intestinal absorption after the oral administration of drugs in humans, whereas lipophilicity was a poor descriptor. Refsgaard et al. [39] found that five descriptors (namely, the number of flexible bonds, number of hydrogen bond acceptors (HBAs) and donors (HBDs), molecular surface area, and PSA) were determinants of permeability in a Caco-2 cell monolayer model. Another study also reported that the most important properties for absorption and permeability were the hydrogen bonding capacity and molecular size of the drug rather than lipophilicity alone [38]. O’Donovan et al. conducted a study using a large in-house dataset comprising >20,000 compounds [40] and identified the molecular descriptors governing bioavailability and membrane permeability in the Caco-2 cell assay. Interestingly, neutral compounds retained permeability up to an MW limit of 700, whereas stronger acids and bases were restricted to a MW of 400–500. 

Existing in silico predictive models for human intestinal absorption (F_a_) are summarized in Table 2. The relationship between the extent of human F_a_ in humans and *P*_app_ in Caco-2 cells with an R of 0.61 was demonstrated by Kamiya et al. [30]. Newby et al. [27] created a computational model to predict human intestinal absorption (HIA; %) from *P*_app_ in Caco-2 cells, and when high and low HIA classes were defined based on a cut-off of 30%, an accuracy of 0.80 was achieved. Various binary classification models have been developed based on data in the literature and/or FDA-approved drugs. The classification model employed a cut-off of 30% in many cases. The classification models by Newby et al. [27] and Shen et al. [41] demonstrated a good predictive performance with sensitivities of 0.741 and 0.998, respectively, and specificities of 0.850 and 85.9–89.7, respectively. In contrast, Niwa et al. [42] produced QSAR models based on a dataset with 86 compounds using a GRNN, even though the dataset was limited.

## 5. Distribution

### 5.1. General Assessment

Predicting the plasma concentration profiles of drugs in humans is important and can be achieved by integrating in vitro and/or in vivo pharmacokinetic data from the nonclinical stages. As a simplified approach, the human profile can theoretically be described by a steady-state distribution volume (V_dss_) in addition to total clearance (CL). The V_dss_ can be predicted by either empirical approaches based on the allometry concept or physiological-based pharmacokinetic (PBPK) models based on the physiological concept. The PBPK approach generally employs mechanistic tissue composition models that are essentially represented by the model described by Poulin and Theil; this approach considers the unbound fractions of the test compound in plasma and tissues. The model described by Rodgers and Rowland [47] considers electrostatic interactions in addition to both unbound fractions. An investigation by Harrell et al. [48] provided insights into drug distribution to the brain and liver; drug distribution to the brain was experimentally determined to be lesser than that estimated theoretically based on in silico- and in vitro-associated data. In contrast, drug distribution to the liver tended to be higher than that predicted by in silico- and in vitro-associated data. Over-prediction in the brain is attributed to some factors, including the involvement of efflux transporters. Berry et al. [49] indicated the usefulness of in vitro nonspecific tissue-binding measurements in predicting V_dss_ for a wide variety of drugs (36 drugs) in rats. Their results implied that the predictive performance based on unbound fractions in plasma and tissues combined with the estimate effect of the pH partition hypothesis was higher than the performance determined using the methods described by Poulin and Theil and Rodger and Rowland [47]. These data highlight the need for in silico direct models based on tissue binding data, including brain tissue and plasma protein binding alongside transporters. There is some debate about the predictability of empirical and in silico approaches for tissue distribution [50], but in any case, it is necessary to improve the predictability of in silico approaches using the latest technologies.

### 5.2. Prediction of Tissue Distribution

In silico linear and nonlinear prediction models of V_dss_ were developed by Berellini et al. [51]. They achieved a relatively high predictive performance; however, over-prediction was an issue for non-steroidal anti-inflammatory drugs. Paixão et al. [52] developed a QSAR model for estimating the tissue-to-blood partition coefficients (K_p,t_) in rats by subjecting K_p,t_ data corresponding to 1460 specimens to an ANN algorithm; the predictive performance was estimated to be 0.909 for the training set and 0.896 for the validation set. Similar models based on QSAR models use multiple linear networks (MLRs). ANNs and SVMs were constructed by Louis et al. [53], who displayed their best predictor with an R^2^ of 0.621. In a unique comparative study—between empirical and in silico predictions of V_dss_—by Fagerfolm et al. [54], no significant difference in predictability was found; 69, 64, and 61% of the prediction within a two-fold error were recognized using rat-to-human scaling, allometric scaling, and the Rodgers-Lukova method, respectively. These methods might be options for researchers wanting to select an appropriate method based on confidence level and/or the costs of running a drug discovery program.

### 5.3. Prediction of Human Plasma (Serum) Protein Binding

Plasma protein binding is one of the key factors considered when carrying out pharmacokinetic, pharmacodynamic, and toxicological evaluations of NCEs. Only the unbound (free) drug is capable of interacting with pharmacological and toxicological targets (receptors, channels, or enzymes), renal glomerular filtration, and hepatic metabolism, and of diffusing between the plasma and organs/tissues. From a pharmacokinetic viewpoint, plasma protein binding affects the volume of distribution (V_d_) and total clearance (CL_tot_) of drugs, which govern their pharmacokinetic profiles [55]. Consequently, for compounds that exhibit high plasma protein binding, small variations in plasma protein binding leads to marked differences in their unbound plasma fraction, which is related to their efficacy and toxicity [56]. Therefore, accurate prediction methods for plasma protein binding are needed for drug discovery and development. 

Binding proteins, such as albumin, α-acid glycoprotein, and lipoprotein, exist in the plasma and serum. Acidic and basic drugs tend to bind albumin and α-acid glycoprotein, respectively. However, albumin and α-acid glycoprotein molecules are rarely utilized in plasma protein binding studies; the exception being in the case of identification studies on drug binding proteins in the regulatory sciences. 

Several in silico prediction models have reportedly been developed (mainly based on QSAR models) to obtain exact predictions of plasma protein binding (Table 3). The utilized datasets can be classified as small (approximately 100 compounds) [54,57] or relatively large (approximately 1000 compounds) [58,59,60]. Global models utilizing datasets comprising 1008 and 1242 compounds spanning a large chemical space were developed by Votano et al. [60] and Zhu et al. [58], respectively; they used a variety of ML methods (RF, SVM, and κ-nearest neighbor [κ-NN]) to predict the bound fraction using the Molconn-Z and Dragon software, respectively. Ingle et al. [59] constructed in silico models with molecular operating environment (MOE) descriptors of 1045 compounds; drugs and environmentally relevant ToxCast chemicals were used to validate their models. Sun et al. [61] developed global models using a pool of molecular descriptors of 967 compounds, which were calculated using PaDEL-descriptors, Schrödinger, and Discovery Studio software. Model performance was evaluated using multifarious validation sets comprising 242 drugs, 397 industrial compounds, and 231 newly designed chemicals. The authors pointed out that the predictive performance of this model was similar to that of high-throughput assays which used corresponding data. 

Small dataset-derived in silico models were created by Fagerholm et al. [54] and Zhivkova et al. [57]. The former study aimed to evaluate the variability in human plasma binding between laboratories for 117 compounds with high plasma binding based on several published reports. It was emphasized in this study that averaged values should be used for compounds with multiple data, as more than ten-fold variability was found for 14 of the 117 compounds. The in silico model developed by PLSR showed a minimum false discovery rate (Q^2^) of 0.69, which was similar to the predictive performance achieved in other studies. The latter study focused on a dataset of 132 diverse acidic drugs with a wide range of binding capacities. An in silico prediction model for f_u,p_ was developed using 178 molecular descriptors based on a genetic algorithm with stepwise regression, and an R^2^ of 0.771 was achieved. As a guide to assess f_u,p_ for acidic compounds, the following checklist criteria were developed: log P ≥ 3, ≥2 aromatic nonconjugated rings, ≥1 cyano groups, ≥3 H-bond donors and acceptors separated by four or five skeletal bonds, ≥1 tertiary C atoms, ≥1 four-membered rings, and ≥1 iodine atoms. Different approaches to optimizing the human serum albumin binding affinity of chemical structures have been proposed. Zhang et al. attempted an in silico docking analysis [62], and identified seven potential high-affinity binding sites of alkylphosphocholine analogues to human serum albumin. The size of the functional groups directly affected the albumin binding and partitioning. Ciura et al. [63] demonstrated the usefulness of ML models using a retention factor in micellar electrokinetic chromatography and chemically advanced template search descriptors based on SMILES, and achieved an R^2^ of 0.869 for MLR and 0.904 for SVM.

### 5.4. Prediction of Brain Distribution

#### 5.4.1. General Assessment of Drug Concentrations in the Brain

The prediction of drug–brain penetration is an essential component when assessing the in vivo pharmacological activities for drugs targeting the CNS [64,65]; this factor also influences CNS toxicity. The BBB in the CNS comprises a continuous layer of endothelial cells joined by tight junctions at the cerebral vasculature. The BBB contains efflux and influx transporters, including P-gp and breast cancer resistance protein (BCRP), which are active efflux transporters that function as barriers for the penetration of drugs across the BBB [66]. 

Drug distribution can generally be defined by the free drug hypothesis, where only drugs without binding to proteins, lipids, or other tissue components can distribute in target tissues; unbound drug molecules are thus able to interact with pharmacological targets [67]. Unbound plasma drug concentrations are generally similar to those in target tissues, with the exception of certain organs like the brain; therefore, the unbound plasma drug concentration is considered to be the pharmacologically effective concentration. In the brain, unbound drug concentrations cannot be deduced from the corresponding plasma concentrations due to the existence of tight junctions, expression of P-gp, and lysosomal trapping of basic drugs [68]. Furthermore, measuring free drug concentrations in the brain is difficult.

Three experimental methods can be used to evaluate drug distribution in the brain, i.e., microdialysis (MD), brain slice, and brain homogenate. The MD method has been utilized to determine the concentration of compounds within the interstitial fluid (ISF) in the brain [69], which enables an estimation of the unbound brain-to-plasma partition coefficient (K_p,uu,brain_) and unbound volume of distribution (V_u,brain_, in mL/g of brain tissue), under the assumption that the unbound drug is in equilibrium between the brain and ISF [70]. This method has been validated with direct evidence of reliable outcomes for drug passage to the brain based on continuous direct sampling. However, the MD method has several issues, i.e., limited utility for lipophilic compounds due to their high adsorption to the MD instrument [71], the necessity for surgical skill, and animal ethics concerns with limited sampling. 

The in vitro brain-slice method (using rats and mice) can yield V_u,brain_ values by measuring the amount of drug present in a brain slice when the drug is incubated with a buffer containing the slice. As the slice maintains the basic structure of the blood and brain tissue, the results obtained using this method tend to account for the influence of nonspecific binding and lysosome trapping. However, this method is not readily replicable in all laboratories because a highly specialized instrument is required for slicing the raw tissue.

The brain-homogenate binding assay estimates the unbound fraction of the drug in homogenized brain tissue (f_u,brain_) with a relatively high throughput. As the brain homogenate contains tissues with ruptured structures, the f_u,brain_ value is likely to be underestimated (compared to the values obtained using the brain-slice method). This underestimation can be attributed to additional binding sites of the drug in the collapsed tissues. f_u,brain_ values are generally evaluated using the brain homogenates of rodents, and there are no reported species differences in f_u,brain_ values.

#### 5.4.2. Prediction of Brain-to-Plasma Concentration Ratio (BBB Permeability)

Many researchers have conducted in vivo brain exposure studies which have yielded total brain-to-plasma concentration ratios, denoted by K_p,brain,_ or log BB (its logarithmic form). Both parameters represent the extent of the compounds passing through the BBB, which is formed by the endothelial cells of the capillaries in the brain. As studies using mice or rats are low throughput due to their labor-intensive and time-consuming nature, many investigators have constructed qualitative or quantitative computational models (classification or QSAR models) and adapted the methods used from MLRs and PLSRs to SVMs and ANNs, following the development of new technologies. 

Lipophilicity, MW, and/or topological PSA (TPSA) are critical parameters used to decide poor or good BBB permeability. A pioneer study by Young et al. [72] demonstrated a relationship between log BB and Δlog P (the difference between log P for octanol/water and log P for cyclohexane/water), which was defined by the hydrogen-binding capacity. Singh et al. [73] summarized the desired physicochemical properties of CNS drugs as follows: MW < 450, AlogP of 1.5–2.5, LogD > 0 and <3, PSA of 60–70 Å^2^, HBAs of <7, HBDs of <3, and RBs of <3. Several QSAR models have been constructed, some of which are introduced here (Table 4). Lanevskij et al. [74] and Gupta et al. [75] each employed three parameters, including log P, pKa, and plasma protein binding; and log P, TPSA, and dipoles, respectively. The latter constructed qualitative and quantitative models using ensemble ML. Chen et al. [76] introduced an in silico model generated using three layers of neural networks with eight descriptors, including high-affinity P-gp substrate probability and plasma protein binding. Vilar et al. [77] proposed two criteria for their classification model, i.e., compounds with log BB > 0.3 pass the BBB readily, whereas compounds with log BB < −1 are poorly distributed to the brain; their respective classifications were defined by log P, PSA, and/or the sum of the number of acidic or basic atoms. This model demonstrated a good predictive performance with >80% accuracy.

Shaker et al. [78] pointed out that the use of prediction models for BBB permeability built on small datasets is usually impractical due to the limited chemical diversity of the compounds. Recently, various classification models for BBB permeability, which are characterized by a high predictivity, have been constructed with relatively larger datasets. Yuan et al. [79] developed binary-classifier models using an SVM with molecular property-based descriptors, including 1D, 2D, and 3D and fragment-based descriptors. A large dataset comprising 5453 compounds for BBB+ and 1709 compounds for BBB- was introduced into a model constructed using a light gradient-boosting machine [81]. Wang et al. [80] were concerned about the imbalance between permeable and non-permeable compounds in the dataset, and resampling methods such as the Synthetic Minority Oversampling Technique (SMOTE) were employed. The resampling method using a SMOTE-edited nearest neighbor effectively solved the imbalanced dataset problem, and a high accuracy of 0.966 was achieved for the external dataset in the final construction of the consensus model; this indicates the usefulness of the SMOTE when utilizing imbalanced datasets. 

Ding et al. [4] reported that a typical ML model is incapable of accounting for the interactions between compounds and proteins, as typified by P-gp-mediated compound efflux. To address this issue, relational graph convolutional networks (RGCNs) were introduced into the construction of in silico models in order to account for these interactions, such as those mediated by ATP-binding cassettes and solute carrier proteins. The RGCN model, which achieved an overall accuracy of 0.872, greatly outperformed the light gradient-boosting machine model. Tong et al. [83] demonstrated the usefulness of uncertainty estimations for improving the predictability of deep learning models for BBB permeability. An uncertainty estimation helps not only to design chemical structures in the lead optimization process, but also to enhance in silico screening for CNS compounds.

#### 5.4.3. Unbound Brain-To-Plasma Partition Coefficient and Brain Homogenate Binding

Based on the free theory concept, the unbound drug concentrations in tissues are generally similar to those in the plasma due to the rapid equilibrium of compounds; however, the K_p,uu,brain_ values in most compounds are not in unity due to the tight junction and barrier system. Therefore, the K_p,uu,brain_ is a crucial parameter for investigating the ability of a drug to penetrate the BBB [84]. Due to the aforementioned limitations of the MD method and animal welfare concerns, it is difficult to directly obtain large amounts of experimental data for K_p,uu,brain_ using in vivo study methods. Several researchers have collected experimental data for f_u,brain_ and V_u,brain_ based on estimates using the brain homogenate and brain-slice methods, respectively. f_u,brain_ and V_u,brain_ are explainable parameters for K_p,uu,brain_, as evidenced via the validation of 81–1121 compounds from the literature or in-house databases [85,86,87,88,89,90]. The in silico models developed for predicting f_u,brain_ and K_p,uu,brain_ are described in Table 5.

Fridén et al. [91] demonstrated that the K_p,uu,brain_ was negatively correlated with hydrogen binding, such as that to PSA or to HBA or HBD. They successfully built a QSAR model using PLSR and 16 descriptors, which achieved a Q^2^ of 0.452. Chen et al. [86] and Varadharajun et al. [87] utilized RF and SVM, respectively, and generated QSAR models with relatively high predictive performances (R^2^ of 0.94–0.96 and Q^2^ of 0.73–0.80, respectively).

**Table 5:** In silico prediction models for unbound compound fractions in brain homogenates and ratio of unbound fraction between brain and plasma.

Parameter	Data Set		Type of Model	Algorithm, Descriptors or Equation of Model	Predictive Performance	Ref.
	No of cpds	Source				
f_u,brain_	470	Experimental data	QSAR	Nonlinear least-squares minimization with log P and pKa	R^2^ = 0.75	[74]
f_u,brain_	2292	In-house data	QSAR	SVM	R^2^ = 0.64	[90]
f_u,brain_	24	Commercial CNS drugs	QSAR	SVM	R^2^ = 0.782	[92]
K_p,uu,brain_	246 for direct model, 173 for indirect model	In-house cpds, research	QSAR	RF, SVM	Indirect model: R^2^ = 0.79–0.9, direct model R^2^ = 0.94–0.96	[86]
K_p,uu,brain_	346	In-house cpds, research	QSAR	RF, SVM	Q^2^ = 0.73–0.80	[87]
K_p,uu,brain_	43	Selected from 92 drugs	QSAR	PLS model, 16 descriptors	Q^2^ = 0.452	[91]
K_p,uu,brain_	640	In-house cpds	QSAR	RF, Conjugate gradient optimization (GPOPT) or incorporation of in vitro data	R^2^ = 0.489 for RF, 0.536 for GPOPT	[93]
K_p,uu,brain_	241	Developmental cpds and marketed drugs	linear regression	A quantum-mechanics-based energy of solvation (E-sol), a linear regression model based on E-sol vs. K_p,uu,brain_ linear	Accuracy = 0.79, R^2^ = 0.61	[94]

The determination of V_u,brain_ from brain slices is one of the approaches for estimating the K_p,uu,brain_ via indirect methods, which was built on a physiological model based on lipid binding and pH partitioning. This model involves the partition of a drug into lipids, ISF, and intracellular compartments of the brain, and the outcomes are in good agreement with experimental data [88]. Similar implicative data of brain slices by Fridén et al. [91] indicated that there were discrepancies between the V_u,brain_ and f_u,brain_, which were attributed to the involvement of pH partitioning in slices with a preserved cellular structure. An indirect method employed by Chen et al. [86] computationally generated K_p,uu,brain_, and achieved an R^2^ of 0.94 for RF and 0.90 for SVM.

Another approach to evaluating the f_u,brain_ is the brain-homogenate method, which has a relatively high-throughput potential in drug discovery. An advantage of this binding assay that no species differences have been observed; the f_u,brain_ showed a one-by-one correspondence curve between mouse and rat brains [92]. A similar result was reported by Di et al. [95], with no species difference in the f_u,brain_ found among seven species, including humans. Thus, the f_u,brain_ in rodents is suggested to be an essential parameter for computational models for predicting the K_p,uu,brain_ in humans, in combination with in silico K_p_ prediction. Lanevskij et al. [74] developed an in silico model for f_u,brain_ using nonlinear least-squares minimization with a dataset of 470 compounds, which had a good predictability with an R^2^ of 0.75. Moreover, in silico models for f_u,brain_ were constructed using SVMs by Wan et al. [92] and Dolgikh et al. [90], both of which demonstrated a relatively high predictive performance with R^2^ vales of 0.782 and 0.64, respectively. Interestingly, Kosugi et al. [93] demonstrated that ML models combined with both in vitro P-gp and BCRP efflux ratios (ERs) yielded predicted K_p,uu,brain_ values that correlated well with in vivo data when compared to ML models. 

#### 5.4.4. P-gp-Related Prediction Models

P-gp, which is a member of the ATP-binding cassette transporter family with a broad substrate specificity, negatively impacts the absorption and distribution of its substrates. This is because P-gp functions as a barrier against xenobiotics in the body. Particularly, P-gp is considered to play a central role in the BBB. Whether NCEs are substrates of P-gp is an important factor when selecting brain-targeting compounds, and some in silico discrimination models have been developed for P-gp efflux. Desai et al. [96] established predictive models based on 2000 structurally diverse compounds, and TPSA and pKa were defined as key criteria for physicochemical property analyses; P-gp substrates with PSA > 60A^2^ and basic pKas < 8 had a decreased distribution. Whether NCEs are subject to P-gp-mediated efflux is generally evaluated using a Caco-2 monolayer or LLC-PK-1 transfected with human P-gp. In the latter case, an ER can be estimated by comparing the P_app_ ratio between Basal (B) to Apical (A) and A to B in LLC-PK-1 cell monolayers with or without over-expressed human P-gp; consequently, P-gp substrates can be defined by net ER ≥ 2, while non-P-gp substrates are defined by net ER < 2. Gunaydin et al. [97] revealed that the following attributes are critical when designing low-efflux compounds: <2 HBD, TPSA of <70 Å^2^, and clogP of >3. In addition, QSAR analyses identified a relationship between net ER (logarithmic scale) and the computationally derived solvation free energy difference, ΔGH_2_O−CHCl_3_. Moreover, Chen et al. [98] developed a QSAR model based on the relationships between descriptors and ERs using HSVR, which achieved an R^2^ of 0.96 for the training set (*n* = 50).

## 6. Metabolism

### 6.1. General Assessment

Drug metabolism studies comprise several components, including the identification enzymes responsible for drug metabolism, the site of metabolism, metabolic inhibition (a drug–drug interaction), and metabolic stability. Among them, metabolic stability can be regarded as the most important parameter governing in vivo pharmacokinetics, which affects in vivo efficacy and toxicity. Thus, the assessment of metabolic stability has been implemented in high-throughput screening (HTS) processes for identifying and optimizing lead structures in drug discovery. Technical supports based on in silico models for metabolic stability have been necessary to reduce the number of screening compounds in HTS with large compound libraries, and guide the direction of structures synthesized during lead optimization. 

In vitro metabolic stability is evaluated using human liver microsomes or human liver S9 fractions. Much attention has been paid to the development of robust in silico models to assess metabolic stability in recent decades. These studies share several common features, i.e., (1) in-house datasets, (2) the use of either intrinsic clearance (CL_int_) [99,100,101,102,103] or half-life (t_1/2_) [104,105,106] as the endpoint of metabolic stability, and (3) in silico classification with mainly binary classifiers of stable or unstable compounds, as shown in Table 6.

### 6.2. Prediction of Microsomal Metabolic Stability

First, we assessed the prediction models developed by major global pharmaceutical companies. Sakiyama et al. [100] developed a binary-clarification model based on CL_int_ using an RF or SVM for a relatively large dataset of 1952 proprietary compounds, which achieved a sensitivity of >0.9 and specificity of >0.6. Large dataset-derived two-classification models were reported by Lee et al. [99] and Gupta et al. [101]. The former used Bayesian statistics and achieved a prediction accuracy of 80%. The latter used a C5.0 decision tree with CDK descriptors, including a set of Smiles Arbitrary Target Specification (SMARTS) keys; this model had a good predictability, and an estimated sensitivity and specificity of 0.57 and 0.91, respectively. These were equivalent to those of models built using commercial MOE2D and the same set of SMARTS keys (a sensitivity and specificity of 0.58 and 0.91, respectively). Binary classifiers are useful for quickly filtering HTS libraries; however, the optimization process may require a moderate category between stable and unstable to retain compounds that are evaluated in the corresponding in-depth assay. Gupta et al. [101] developed three-classification models (CL_int_) that categorized low (CL_int_ < 9.2 μL/mg/min), moderate (9.2 < CL_int_ < 48), and high (CL_int_ > 48) risk, and had a high predictive performance shown by RF or SVM.

This relatively high predictability might be due to the use of standardized protocols that ensure consistent data in each individual laboratory. Sasahara et al. employed an intriguing prediction strategy [102,103]. Their first approach was to set up the balanced dataset with stable and unstable compounds (50.9 and 49.1%, respectively). Second, when the compounds to be predicted were outside the applicability domain (AD) on the in silico model, the predictive results were unreliable, thereby possibly misleading future compound design. Therefore, a reliable prediction model to evaluate the AD consistency of newly designed compounds was developed, which, consequently, resulted in the prediction accuracy increasing from 0.799 to 0.936. 

Many researchers have employed t_1/2_, which can be directly determined as an endpoint in substrate depletion assays. Liu et al. [104] and Li et al. [106] constructed in silico models with high predictive performances based on t_1/2_ using datasets from ChEMBL, an open database. The models by Liu et al. of human metabolic stability achieved a sensitivity of 0.73–0.78 and specificity of 0.85–0.88 for the nearest neighbor. The in silico predictive models by Li et al. yielded an AUC of 0.84 for rat metabolic stability and 0.86 for human metabolic stability. Furthermore, the National Center for Advancing Translational Sciences aimed to build global and local models to accommodate for the large and unique dataset of >24,000 compounds from more than 250 projects that covered a wide range of pharmacological targets and biological pathways [105]. The local models were built for projects which performed poorly on the global model. The global predictive model is freely accessible for any researcher (https://opendata.ncats.nih.gov/adme (accessed on 6 November 2023)). However, t_1/2_-based models have a disadvantage with respect to applicability relative to CL_int_-based models. The t_1/2_ parameter depends on experimental conditions such as microsomal concentrations and incubation time in metabolic stability studies; in contrast, CL_int_ is normalized in terms of microsomal concentration and incubation time. Thus, similar to CL_int_-based models, the predictive outcomes from t_1/2_-based in silico models provide insights into chemical structure design; however, recalculation based on differences in experimental conditions is required. 

### 6.3. Prediction of Total Clearance

An alternative computational approach is to directly predict total clearance. When elimination from the body only occurs hepatically, the hepatic clearance is equal to the total clearance. Berellini et al. [107] developed an in silico PLSR model to predict human plasma clearance based on a large dataset comprising 754 compounds using physicochemical descriptors and structural fragments. Validation of this model was performed using the Enhanced Leave Analog-structural, Therapeutic, Ionization Class Out approach based on ten therapeutic or structural analog classes. The model gave a percentage of compounds within two- and three-fold errors of 59% and 80%, respectively. Another approach based on the concept that analog compounds display similar pharmacokinetic properties [108] predicted the total clearance by comparing the known reference compounds of four chemical classes, including acids, bases, zwitterions, and quaternary ammonium/pyridinium ions using κ-NN. The prediction accuracy averaged within two-fold errors for all the chemical classes in the κ-NN model and outperformed two allometric approaches typically used for human total clearance prediction, even with a limited number of data (18 drugs). In contrast, a PLSR model achieved a quantitative performance of the total clearance comparable to that of animal scaling based on the allometric concept [109]. Interestingly, Kosugi and Hose [110] demonstrated the great usefulness of the ML approach in terms of predicting total clearance, which is relative to the in vitro–in vivo extrapolation (IVIVE) method based on a well-stirred model with experimentally obtained CL_int_. The total clearance values predicted by the RF and radial basis function models were within two-fold of the observed values for 67.7 and 71.9% of the test set compounds, respectively; both models also showed potential for improvement via the incorporation of in vitro parameters such as f_u,p_ and CL_int_. It should be noted that the predictability of the in silico approach based on total clearance leads to the possibility of a paradigm shift occurring with respect to the metabolic stability-based optimization process in the early stages of drug discovery.

### 6.4. Molecular Modeling and Simulation

**Scheme 1:**
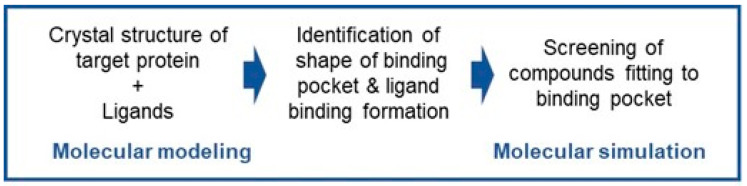
Compound screening using molecular modeling and simulation.

With recent scientific and technological advances, protein structures have been widely used to predict the binding modes of ligands or designed compounds in docking algorithms for rational drug design. As shown in Scheme 1, molecular modeling and simulations based on target protein structures have become an important in silico screening tool for identifying lead compounds in drug discovery [111]. As the crystal structures of proteins are elucidated, pharmacokinetic researchers are adopting molecular modeling and simulation methods for when conventional in silico prediction using ML fails. 

CYP3A4, a major drug-metabolizing enzyme, is involved in the metabolism of 30% of the drugs in current use [112]; this can be attributed to its binding pocket being larger [113] and more flexible [114] than that of the other CYPs. Sato et al. elucidated the crystal structures of several other proteins—similar to CYP3A4—for the pre-training and subsequent fine-tuning of a CNN-based model [115]. The effects of different datasets on the prediction of the binding mode of CYP3A4 were investigated using a three-dimensional neural network. It was revealed that a dataset with a large median binding pocket size may be important for the binding model prediction of CYP3A4. Such molecular modeling approaches have been employed in the characterization of various metabolic profiles [116]. This may help researchers to design NCEs with efficient metabolic profiles for various classes of therapeutic targets.

## 7. Renal Excretion

### 7.1. General Assessment 

Renal clearance refers to the excretion of unchanged drugs through the kidneys and represents the major elimination route for drugs with negligible metabolism and biliary excretion. Renal excretion is an integrated process that comprises glomerular filtration, tubular secretion, and reabsorption [117]. Glomerular filtration describes the ultrafiltration of approximately 10% of the total renal blood flow at the glomerulus of the nephron, which leads to an average glomerular filtration rate (GFR) of 125 mL/min in a young man weighing 70 kg. As only unbound drugs undergo glomerular filtration, glomerular filtration clearance is theoretically equal to the product of GFR and f_u,p_ [118]. Tubular secretion is facilitated by several efflux transporters that are predominantly expressed in the proximal tubule. In contrast, reabsorption mainly involves passive permeability that is dependent on urine flow, pH, and some influx transporters, which are localized at the proximal tubules. Thus, net secretion or net reabsorption is apparent when renal clearance is greater or less than f_u,p_*GFR; however, both secretory and reabsorption processes sometimes occur simultaneously to different extents.

Both renal clearance (CLr) and the fraction of compounds excreted in their unchanged form in the urine (fe) are important predictive indicators for renal drug excretion. CLr is a governed parameter for pharmacokinetic profiles and is calculated using the proportionality term between the urinary excretion rate and plasma concentration of unchanged compounds [117]. The fe value of unchanged compounds can theoretically be decided by the balance between hepatic clearance and CLr; however, estimating these parameters in humans is difficult. The fe value is experimentally determined through human mass balance studies after the intravenous administration of radio-labeled drugs during the clinical stage. To predict CLr, allometric scaling and IVIVE approaches have been extensively utilized. However, allometric scaling, a practical measure, requires in vivo CLr data in several animal species [119], and acquiring these in vivo data is a labor-intensive and time-consuming process. IVIVE approaches have been successfully utilized, but in vitro, permeability data from Caco-2 or LLC-PK1 cells are needed for these models [118]. Accordingly, the establishment of computational approaches is valuable for estimating both parameters. Table 7 summarizes the existing in silico prediction models for renal clearance and urinary excretion.

### 7.2. Prediction of Renal Clearance

Doddareddy et al. [120] developed QSAR models for predicting CLr using the PLSR algorithm with 130 marketed drugs. The predictive performance achieved an R^2^ of 0.844 and 0.720 when using Volsurf and Molcom-Z descriptors, respectively. Chen et al. [121] generated local and global models for predicting CLr. The local models (based on stepwise multiple linear regression and RF) were generated for their respective compound classes; namely, compounds exhibiting different ionization states such as acid, bases, and neutral compounds; or compounds exhibiting the pharmacokinetic behaviors of net reabsorption and net secretion. Global models based on various approaches, such as ANN, classification trees, κNN, RF, and SVM, were generated for all investigated compounds. A similar QSAR model [122] was able to predict net secretion clearance and net reabsorption clearance with a Q^2^ of 0.77 and 0.81, respectively. Furthermore, it was pointed out that f_u,p_ was a key parameter in CLr prediction, which may be explained by the fact that unbound compounds only undergo filtration and secretion in the kidney.

### 7.3. Prediction of the Fraction of Urinary Excretion

As reported in the prediction of absorption fraction, the proportion of urinary excretion is usually discussed qualitatively, leading to the development of classification approaches based on the excretion fraction criterion. Criteria setting is likely to differ between researchers. Kusama et al. [123] computationally developed several ADMET prediction models based on 141 approved drugs and adopted a criterion of 50% for fe; fe > 50% was regarded to indicate renally excreted compounds. Classification models for fe were constructed based on the rectangular method with the f_u,p_, MW, and log D of 41 drugs, and the predictive performance was estimated to be 90% for recall. Similar investigations were conducted by Toshimoto et al. [124] and Wakayama et al. [125]. Wakayama et al. enlarged the chemical space of the compounds by increasing the number of compounds in the dataset, and employed SVM and a two-step approach with subset clustering; they reported a similar predictive performance to that of Kusama et al.’s model. Noticeably, a prediction model generated by Doddareddy et al. [120] utilized a criterion of 20% for renally excreted compounds. They generated a binary classification model from structural information calculated using Volsurf and Molconn-Z, with the threshold value of fe set to 0.2 in a dataset containing 130 compounds. This resulted in 65–80% of all test sets being correctly predicted. Cut-off values, which are important for classification models, should be set depending on what pharmacokinetic characteristics are identified as being crucial in the drug discovery strategy.

## 8. Prediction of Transporter Substrates Involved in ADME

Two major families of transporters, ATP-binding cassette (ABC) transporters and solute carrier (SLC) transporters, are known to govern the pharmacokinetic profiles of their substrates. Multidrug-resistance-associated proteins (MRP2/3/4) (in addition to P-gp and BCRP, which are described in “Section 5.4”) belong to the ABC transporter family, and organic anion-transporting protein (OATP)1B1/1B3, organic anion transporter (OAT)1, OAT3, organic cation transporter (OCT)1/2, and multidrug and toxin extrusion (MATE)1/2-K belong to the SLC transporter family. These transporters are located in polarized cells in the intestine, liver, kidney, and/or BBB, and significantly affect ADME. When evaluating development compounds and candidates, it is necessary to examine whether they are substrates for any of these transporters. This requires in vitro systems with cells expressing each transporter, and is time-consuming and expensive. Accordingly, in silico predictive models to determine if a compound is a substrate of these transporters are desired, however, few in silico models have been created, possibly due to a limited data available in public databases.

Kusama et al. attempted to perform an in silico prediction of hepatic uptake via OATPs, and employed rectangular boxes with MW, log D, and f_up_; they achieved a recall of approximately 0.67 [123]. Toshimoto et al. performed a similar prediction for hepatic uptake via OATPs, with a recall of 0.78, which was higher than that of the rectangular method [124]. Importantly, Ose et al. developed an SVM-based system to evaluate whether a given compound is a substrate of seven categories of drug transporters, which included OATP1B1/1B3, OAT1, OAT3, OCT1/2, MATE1/2-K, P-gp, and BCRP [126]. Four physicochemical parameters (MW, log D, f_up_, and charge) were utilized as basic parameters. Much effort by the author’s group was made to compile a negative dataset for non-substrates of each transporter, owing to the paucity of data in public databases. Expert-involved curation was conducted to generate a dataset of putative non-substrates for each transporter based on the personal opinions of 11 researchers in the field. The developed model correctly predicted 111 of 136 compounds as substrates for an external dataset. This information will be useful for the prediction of renal clearance by in silico models because SCL transporters are often involved in renal excretion processes.

## 9. Toxicity

The safety of NCEs in humans must be ensured based on results from various toxicological studies during drug discovery. Recently, ML has become increasingly prominent in predictive toxicology as toxicological assessments have shifted from in vivo studies to in silico studies via in vitro studies. Various models have been used to predict toxicological endpoints. Wang et al. summarized in silico models to predict various toxicity endpoints, such as cardiotoxicity, hepatotoxicity, genotoxicity, immunotoxicity, acute oral toxicity, and developmental toxicity via various ML modalities [127]. However, these ML approaches achieved AUCs of 75% or above, which was lower than expected. Therefore, much effort is needed to address the main bottleneck facing ML in predicting toxicological events, which is to ensure the quality and quantity of data in the training datasets. Ogura et al. indicated that most researchers constructed their datasets from only one database, consequently leading to a limited predictability [128]. An integrated dataset (comprising over 291,000 structurally diverse compounds derived from ChEMBL, GOSTAR, and PubChem) successfully resulted in an outperforming classification model for hERG inhibition via the use of a SVM with descriptor selection based on the Non-dominated Sorting Genetic Algorithm-II.

The importance of curation when creating high-quality training datasets was demonstrated by Alves et al., who showed that the inclusion of a large number of duplicated data in data repositories artificially yielded a high predictability in the case of no curation [129]. The curation process is essential for developing robust and reliable predictive in silico models to evaluate the toxicological profiles of compounds. Imbalances between positive and negative data in training datasets are a critical issue. Recently, Hu et al. employed a chemprop model which combined a Directed-Message Passing Neural Network module for graph-based molecular representation and a feedforward neural network module for classification or regression [130]. The chemprop models performed best when predicting carcinogenicity, cardiotoxicity, developmental toxicity, hepatotoxicity, nephrotoxicity, neurotoxicity, and reproductive toxicity endpoints.

The goal in toxicological evaluations has shifted from evaluating toxicity alone to mechanistically explaining any toxic outcomes, which allows researchers to improve the safety profiles of drugs [131]. In general, in silico models are developed to predict two major toxicological events: first, the direct interaction of the compound with cellular organelles or compartments; and second, the resulting apical endpoint, which is commonly used for hazard evaluation. The first is termed the molecular initiating event (MIE), and the second is termed the adverse outcome. A cascade from the MIE to the adverse outcome via key events is referred to as an adverse outcome pathway (AOP). AOPs can be used for all compounds which are associated with available mechanistic evidence, and the AOP framework is a powerful tool for bridging the gaps between in silico disciplines. Currently, numerous AOPs are under development for a range of complex toxicity endpoints. Based on AOPs, respective ML models for MIE(s) or key events will help to guide decision making in drug discovery. Furthermore, following an in silico identification of MIE, toxic outcomes for MIE can be extrapolated to potential in vivo toxicity using a PBPK model drawing of the plasma concentration profile [132]. Given that plasma concentrations can be predicted by integrated in silico predictive models for ADME, integrated in silico approaches for ADMET can computationally describe toxicological outcomes.

## 10. In Silico Prediction Models Applicable to Academic Research

There are several commercial software packages available for predicting ADME parameters, including ADMET Predictor, BIOVIA Discovery Studio, and SCIQUICK. These suites are widely used in the pharmaceutical industries during the early stages of research and development. Furthermore, in silico ADME models constructed by various researchers have been based on datasets comprising in-house data from institutes or data extracted from commercially available databases, and the calculation tools of descriptors and algorithms in the commercially available software are utilized. This can be attributed to several reasons; this increases the predictive performance of in silico models, utilizes reliable software (which has been validated) for both calculating the descriptors and producing the prediction model, and constructs a model using a dataset selected from a chemical space congruent to its own library. 

However, high licensing fees for those packages preclude their use by academic researchers; therefore, the construction of a well-established environment for using such prediction tools is essential for academic drug discovery. To meet such a demand, freely available prediction models have been developed, including OChEM [8], SwissADME [9], pkCSM [10], and ADMETlab 2.0 [133,134]. OchEM is a web-based platform with two major subsystems, a database of experimental measurements and a modeling framework. SwissADME is a new web tool that allows free access to a pooled robust predictive model for physicochemical properties, ADME, and drug-likeness with medicinal chemistry friendliness. pkCSM utilizes graph-based signatures to develop predictive models, and its web tool provides predictive models for ADME and toxicological properties. ADMETlab 2.0, which is a completely redesigned version of the widely used AMDETlab web server, enables the prediction of pharmacokinetic and toxicity properties, including 17 physicochemical properties, 13 medicinal chemistry properties, 23 ADME properties, 27 toxicity endpoints, and 8 toxicophore rules. The AMED established the iD3-INST with financial support, with the goal of constructing a drug discovery platform with the components of a high-quality open-access database including public data and in silico prediction models for ADME, cardiotoxicity, and a drug-induced liver injury model [11,135]. 

Our group compiled ADME data from ChEMBL (http://www.ebi.ac.uk/chembl/ (accessed on 6 November 2023)) and manually processed the data by checking the experimental protocols, values, units, and other details. The experimental in-house data for some parameters were obtained under unified conditions. Moreover, the iD3-INST originally acquired the experimental data regarding the net ER in P-gp-overexpressed LLC-PK1 cells. Using integrated publicly available databases, in silico prediction models were developed for the following nine parameters: solubility, membrane permeability, HIA [136], human plasma protein binding [137], brain homogenate binding [138], net ER involving P-gp [139], metabolic stability [140], urinary excretion, and renal clearance [141]. The characteristics of these models are summarized in Table 8. The prediction models were constructed using descriptors calculated with free software such as PaDEL, CDK, jCompoundMapper, Rdkit, and Modred, and algorithms such as RF, LightGBM, SVM, and gradient boosting. 

Despite our use of free software and public data, our in silico models appeared to yield predictive performances comparable to those of the reported models that were based on in-house databases or commercially available tools. Particularly, some in silico models were created based on the pharmacokinetic screening aspect of drug discovery. In general, in silico models for plasma protein binding allow for the prediction of a range of binding or unbound fractions. However, NCEs tend to be highly lipophilic and often exhibit a high binding affinity to plasma proteins; therefore, to address this issue, in silico models have been generated using compounds with unbound fractions (f_u,p_) less than 0.1. Furthermore, predicting brain distribution may require in silico models to assess P-gp efflux against screening compounds. Much effort has been made to obtain data regarding net ER by comparing P_app_ in LLC-PK1 cells with and without P-gp overexpression, thereby successfully allowing for the development of a three-classification model for ER with a relatively high predictive performance [142]. Consequently, this model allowed us to develop a mechanistic model for predicting brain penetration, which considers P-gp and net ER [139]. A series of prediction models and integrated databases have been placed in a web-based open-access platform: Drug Metabolism and Pharmacokinetics Analysis Platform (DruMap; https://drumap.nibiohn.go.jp/ (accessed on 6 November 2023)).

An informatics public–private partnership (iP3) consortium, comprising public (NIBIOHN and RIKEN) and private sectors (seven Japanese pharmaceutical companies or divisions) which hold high-quality data, was organized by the iD3-INST. With the dedicated support of AMED, discussion at the iP3 consortium has led to an agreement for a blueprint. Based on the blueprint, the iD3-INST received data on solubility, human plasma protein binding, and metabolic stability from the private sectors and also obtained data for net ER using compounds provided by the private sectors [143]. The establishment of the PP database by integrating private data in a public database led to the creation of seven in silico prediction models for solubility, f_u,p_ in humans, f_u,p_ in rats, net ER, f_u,brain_, CL_int_ in humans, and CL_int_ in rats [11]. These in silico models have been made available for academic drug discovery and incorporated into SCIQUICK to build an ecosystem that maintains the iD3-INST integrated platform. 

In August 2020, iD3 launched the “Development of a Next-generation Drug Discovery AI (DAIIA) through Industry-Academia Collaboration” initiative, which aims to improve the efficiency of drug discovery in academia and efficiently create NCEs from drug discovery research in industry. DAIIA aims to develop a next-generation drug discovery AI platform via industry–academia partnership, and has combined data on the binding affinity between drug discovery target biomolecules and compounds held by both industry and academia. Furthermore, empirical knowledge on structural optimization held by drug discovery chemists in industries and other data from multiple aspects of drug discovery research have been shared in the partnership. Nearly 18 member industries of the Japan Pharmaceutical Manufacturers Association Research and Development Committee are participants in the DAIIA project, and the full-scale operation of the industry–academia partnership is ongoing. By using leading-edge AI technologies, DAIIA is developing predictive models for compound design, binding affinity, and ADMET, which will have practical applications in the field of drug discovery for both industry and academia. 

## 11. Future Prospective and Conclusions

To address issues regarding the predictability of ADME profiles, many researchers have improved upon existing computational ADME models with the advent of AI, and, recently, graph convolutional networks have been equipped with the ability to predict protein functions by exploiting sequence features extracted from a protein language model and protein structures [4]. The predictive accuracy of existing in silico models has been comparatively evaluated, as shown by the statistical values reported in the literature; however, the datasets utilized in individual studies often differ due to different source data, including in-house generated data, published data from the literature, or public data extracted using an origin-curation method. A limited number of studies have performed comparative assessments based on the same test set to objectively evaluate the predictive accuracy between in silico models. Except for these cases, at present, the determination of the superiority or inferiority of specific in silico models remains difficult.

Some research has suggested an alternative evaluation approach for predictive performance in in silico prediction models toward relevant in vivo parameters, such as CL and CLr; outcomes from the in silico prediction are evaluated by comparing the corresponding data estimated using the allometric and IVIVE methods. These methods are based on the allometry concept and physiological modeling, respectively. This trend has emerged due to unsatisfactory results from predictive models for in vivo human parameters, such as CL and V_dss_, via allometric (using relevant animal data) or IVIVE (using in vitro experimental data) approaches. 

Other investigations have suggested that predictions of in vivo pharmacokinetic profiles can be assessed by integrating not only various in silico-based parameters, such as P_app_, CL_int_, and f_u,p_, but also in vitro experimental data and in silico-derived parameters in a physiologically based pharmacokinetic model [144,145]. Unique investigations by Kosugi and Hose [110] demonstrated the usefulness of a combination of in silico descriptors and in vitro ADME properties for predicting in vivo oral exposure, evidenced by the better predictability of the combination method when compared to the IVIVE or empirical methods. Similar efforts were made by Miljković et al. [146] toward predicting in vivo pharmacokinetic parameters. Recent advances in in silico prediction appear to expand to in vivo pharmacokinetics profiles related to efficacy and toxicity. A unique ML model that successfully predicted the plasma concentration time profiles after intravenous or oral dosing in 17 in-house projects was developed [147]. This provides insights into the optimization approaches that can be used in in silico models to generate satisfactory predictive tools which directly draw on in vivo pharmacokinetic profiles, in addition to predicting individual in vitro parameters to guide the direction of chemical synthesis. Additionally, Iwata et al. [148] focused on the fact that there are missing data in nonclinical studies and, therefore, increased the number of training compounds and nonclinical datasets by performing missing-value imputation and feature selection on nonclinical data. Novel models were successfully constructed for CL and V_dss_ with high prediction accuracies. 

This review highlights the innovative technologies that have been incorporated into the field of in silico ADME prediction. Furthermore, in silico modeling is expanding from the prediction of individual in vitro parameters in drug discovery to in vivo pharmacokinetic prediction in nonclinical studies. In silico models are utilized in a wider range of areas than before, and are becoming a crucial tool in drug research and development. In the future, sharing pharmacokinetic and toxicological data in clinical trials between industries and academia will allow academia to develop more useful models for human applications.

Given that academic research occupies a central position in making breakthroughs in drug discovery, it is important that future models support academic drug discovery. AMED launched two projects to improve the academic drug discovery environment by constructing a platform consisting of an open-access database and software package, and the models showed acceptable predictive performances. This platform will enable the acceleration of the production of NCEs with desirable profiles (including ADME) in academic drug discovery. Future efforts are required to continue integrating high-quality screening data from industries and cutting-edge technologies from academia. The expansion of similar initiatives is expected to strengthen the AI-based environment of computational ADME prediction in the academic drug discovery field. Finally, the prediction of in vivo pharmacokinetic profiles rather than individual ADME parameters will become a useful tool for academic drug discovery researchers who may have limited resources.

## Figures and Tables

**Table 1:** In silico prediction models for solubility and membrane permeability in cell-based assays.

Assay	Dataset			Type of Model	Algorithms	Predictive Performance	Ref.
	No of cpds	Source	Parameters				
Solubility	483	AQUASOL and SRC databases	Aqueous solubility	Two-classification model	Cart classification model	Sensitivity = 0.823, selectivity = 0.879	[27]
Solubility	11,780–22,209	In-house assay data	Kinetic solubility	Two-classification model	RF, GCNN	AUC-ROC = 0.87–0.90, Sensitivity = 0.63–0.71, Specificity = 0.90–0.91	[28]
Solubility	12,674	Two public databases	Aqueous solubility	Combination of two-classification and regression models	Gradient boosting and recursive RF	R^2^ = 0.87 for consensus model Sensitivity = 0.80, specificity = 0.96	[29]
Permeability	207	In-house cpds measured	Caco-2 cells	QSAR	Multivariant linear regression	R = 0.76	[30]
Permeability	130	Literature	Caco-2 cells	QSAR	Innovative machine learning-based HSVR	R^2^ = 0.91	[31]
Permeability	386	Marketed drugs	MDCK cells	QSAR	PLS, SVM	AUC = 0.84 for PLS, 0.81 for SVM	[32]
Permeability	74	Discovery cpds	Rat intestinal permeability	QSAR	HSVR	R^2^ = 0.93	[33]
Permeability	71	Drugs peptide mimic compounds	PAMPA	QSAR	Regression analysis	R^2^ = 0.76	[34]
Permeability	182	Literature	PAPMA	QSAR	PLS, HSVR	Q^2^ = 0.88 for HSVR and 0.61 for PLS	[35]
Permeability	>6500	Drugs and drug-like compounds	PAMPA	Four-classifier models	Graph convolutional neural network	Sensitivity = 0.74 specificity = 0.82	[24]
Permeability	2406–16,624	In-house	PAMPA	Two-classifier model	RF, GCNN	AUC-ROC = 0.85–0.86, Sensitivity = 0.83–0.84, Specificity = 0.64–0.78	[28]

**Table 2:** In silico prediction models for human intestinal absorption.

Assay	Dataset			Type of Model	Algorithms	Predictive Performance	Ref.
	No of cpds	Source	Parameter				
HIA	86	Drugs and drug-like cpds	F_a_	QSAR	GRNN, probabilistic neutral network	RMS = 22.8% for GRNN,	[42]
HIA	455	Drugs and drug-like cpds	F_a_	QSAR, Two-classifier model	Genetic function approximation, recursive partitioning techniques	R = 0.84, 95.9% poorly absorbed compounds and 96.1% well-absorbed compounds	[43]
HIA	141	ChEMBL, research and serotonin database	F_a_	Two-classifier model, QSAR	Hierarchical combination of classification and regression	Accuracy = 0.765, precision = 0.782 R^2^ = 0.379	[44]
HIA	932	Several research and FDA drug databases	F_a_	Two-classifier model	Cart classification model	Sensitivity = 0.745, specificity = 0.865	[27]
HIA	578	Hou’s research	F_a_	Two-classification model	SVM	Sensitivity = 0.998, specificity = 0.859–0.897	[41]
HIA	225	Zhao’s research	F_a_	Classification models	Gaussian process classification, RF, SVM	Gaussian process classier RF, SVM κ = 0.42–0.58	[45]
HIA	578	Research	HIA	SAR-based SAR model, QSAR	Linear SAR and ensemble learning-based SAR modeling	Qualitative SAR > 99%, QSAR: R^2^ > 0.91	[46]

**Table 3:** In silico prediction models for plasma protein binding.

Assay	Data Set			Type of Model	Algorithms	Predictive Performance	Ref.
	No of cpds	Source	Parameter				
Human plasma protein binding	117	Data from the literature	Unbound fraction	QSAR	PLS	Q^2^ = 0.69	[54]
	132	Obach’s database	Unbound fraction	QSAR	Stepwise linear regression	R^2^ = 0.771, Q^2^ = 0.737	[57]
	1045	Drugs	Unbound fraction	QSAR	RF, SVM, κ-nearest neighbor	MAE = 0.10–0.18	[59]
	1242	Drugs or drug-like cpds from DrugBank, etc.	Bound fraction	QSAR	RF, SVM, κ-nearest neighbor	R^2^ = 0.67	[58]
	967	DrugBank, etc.	Bound fraction	QSAR	RF, SVM, κ-NN, multi-layer NN	MAE = 0.129–0.178	[61]
	1008	Experimental data	Bound fraction	QSAR	MLR, artificial neural network, SVM	MAE = 7.6–18.3, R^2^ = 0.61–0.90	[60]

**Table 4:** In silico prediction models for evaluating the ratio between brain and plasma concentrations (BBB permeation).

Assay	Data Set		Type of Model	Algorithm, Descriptors or Equation of Model	Predictive Performance	Ref.
	No of cpds	Source				
BBB permeation	470	Experimental data under steady-state condition	QSAR	nonlinear least-squares minimization with logP, pKa, plasma protein binding	R^2^ = 0.52	[74]
BBB permeation	362	In vivo data from some research	QSAR	Nonlinear model with XlogP, TPSA, Dipole	R^2^ = 0.926	[75]
BBB permeation	120	In vivo data	QSAR	Three layered feedforward NN	R^2^ = 0.67	[76]
BBB permeation	307	In vivo data from some publications	QSAR	Model 1: log BB > 0.3 log BB class = 0.5159 × log P(o/w) − 0.0277 × TPSA − 0.3462 Model 2: log BB < −1 log BB = 0.2289 × logP(o/w) − 0.0326 × TPSA−0.5671 × (a.acid + a.base) + 2.3420	Good classification > 0.80	[77]
BBB permeation	1147	The literature and world drug index dataset	Two-classification model	RF, MLP, and SMO (sequential minimal optimization)	Consensus model Accuracy = 0.88, selectivity = 0.88, specificity = 0.88	[73]
BBB permeation	7162	7 studies	Two-classification model	Light gradient-boosting machine	Selectivity = 0.90, specificity = 0.94	[78]
BBB permeation	1990	In vivo data from some research	Two-classification model	SVM with molecular property-based descriptors including 1D, 2D and 3D descriptors and fingerprints	Selectivity = 0.962, specificity = 0.944 and Q^2 =^ 0.957	[79]
BBB permeation	2358	In vivo data from some research	Two-classification model	SVM with MACCS fingerprints	Accuracy = 0.966	[80]
BBB permeation	3961	The literature	Two-classification model	Relational GCN	Accuracy = 0.872, sensitivity = 0.919, specificity = 0.763	[81]
BBB permeation	2342	The literature	Two-classification model	Deep-learning-based recurrent neural network model	Accuracy= 0.965, selectivity = 0.949, specificity = 0.981	[82]
BBB permeation	18	PAMPA	QSAR	Stepwise MLR, PLS, SVM	R^2^ = 0.86 for MLR, 0.73 for PLS, 0.81 for SVM	[83]

**Table 6:** In silico prediction models for evaluating metabolic stability in human and rat liver microsomes.

Assay	Dataset			Type of Prediction Models	Algorithms	Predictive Performance	Ref.
	No of cpds	Source of cpds	Parameter				
Human liver microsome	14,557	Cpds from various laboratories within company	CL_int_	Two-classification model	RF, Bayesian	Prediction accuracy = 0.80	[99]
Human liver microsome	1952	Proprietary cpds	CL_int_	Two-classification model	RF, SVM	Sensitivity > 0.9, specificity > 0.6	[100]
Human liver microsome	49,968	Synthesized cpds by in-house projects	CL_int_	Three-classification model	RF, SVM C5.0 decision tree	Sensitivity = 0.57, specificity = 0.91	[101]
Human liver microsome	26,138	Proprietary cpds	CL_int_	Two-classification model	RF, XGB, GCN	Accuracy = 0.799	[102,103]
Human liver microsome	4012	ChEMBL	t_1/2_	QSAR Classification model	RF, variable nearest neighbor	Sensitivity = 0.78, 0.73, specificity =0.85, 0.88	[104]
Rat liver microsome	>24,000	Cpds from > 250 projects at NCATs	t_1/2_	Two-classification model	RF, deep neural network, GCNN	Sensitivity = 0.86, specificity = 0.77	[105]
Human and rat Liver microsome	4771 for humans, 2512 for rats	ChEMBL	t_1/2_	Two-classification model	RF, XGBoost, neural network, κ-nearest neighbor	AUC = 0.86 for human, AUC = 0.84 for rat	[106]

**Table 7:** In silico prediction models for renal clearance and urinary excretion.

Parameters	Data Set		Type of Model	Algorithms	Predictive Performance	Ref.
	No of cpds	Source				
CLr	130	Marketed drugs	QSAR	PLS	R^2^ = 0.844 by Volsurf; R^2^ = 0.720 by Molcom-Z	[120]
CLr	250	Drugs and drug-like compounds	Two-classify-cation model (distinguish GNetR and GNetS)	ANN, classification tree, κ-nearest neighbor, RF, SVM	AUC = 0.66–0.68	[121]
CLr	636	Drugs and drug-like compounds	Global model	MLR and RF	R^2^ = 0.21–0.36	[121]
CLr	265–371	Drugs and drug-like compounds	Local model (GNetS, GNetR)	MLR and RF	R^2^ = 0.45–0.54 for GNetS, R^2^ = 0.48–0.76 for GNetR	[121]
CLr	382	Drugs and drug-like compounds	QSAR	StepwiseMLR	R^2^ = 0.79 for net reabsorption clearance	[122]
Renal elimination	141 (Renal:41)	Approved drugs	Two-classification model	Rectangular approach (3-dimentional analysis with fu,p, MW and log D)	Recall = 0.90	[123]
Renal elimination	141 (Renal:41)	Approved drugs	Two-classification model	Rectangular approach, SVM	Recall = 0.71–0.90	[124]
Renal elimination	419	Drugs	Two-classification model	SVM, single-step approach, two-step approach with subset clustering	Recall = 0.85	[125]

**Table 8:** In silico prediction models constructed by our group for academia drug discovery.

Pharmacokinetic Items	Dataset (No of cpds)	Type of Models	Algorithms	Predictive Performance	Ref
Solubility	367	Two-classification	Linear SVM	Accuracy: 0.811, Kappa: 0.628	[136]
Permeability	3532	Two-classification	Linear SVM	Accuracy: 0.824, Kappa: 0.401	[136]
Intestinal absorption	946	Three-classification	Linear SVM	Accuracy: 0.836, Kappa: 0.560	[136]
Plasma protein binding	2738	Regression	RF	R^2^ = 0.691	[137]
Brain homogenate	253	Regression	Gradient boosting	R^2^ = 0.630	[138]
P-glycoprotein: net efflux ratio	28–46	Confusion matrix model	Gradient boosting	Kapp = 0.45	[139]
Metabolic stability	4685	Three-classification	Radial SVM	Accuracy: 0.771 Kappa: 0.588	[140]
Urinary excretion	411	Two-classification	RF	Balanced accuracy: 0.74	[141]
Renal clearance	401	Two-step prediction system: three-classification and regression model	RF	R^2^ for reabsorption type: 0.47, intermediate type: 0.68, secretion type: 0.46	[141]

## Data Availability

The data can be shared up on request.
